# A Pan-Cancer Analysis of Heat-Shock Protein 90 Beta1(HSP90B1) in Human Tumours

**DOI:** 10.3390/biom12101377

**Published:** 2022-09-26

**Authors:** Yaxuan Wang, Xiaolin Wang

**Affiliations:** 1 Department of Medicine, Nantong University, Nantong 226000, China; 2 Department of Urology, Affiliated Tumor Hospital of Nantong University (Nantong Tumor Hospital), Nantong 226361, China

**Keywords:** HSP90B1, cancer, prognosis, methylation, phosphorylation

## Abstract

Background: HSP90B1, a member of the heat-shock protein 90 family, plays a vital role as a molecular chaperone for oncogenes and stimulates tumour growth. However, its role in various cancers remains unexplored. Methods: Using the cancer genome atlas, gene expression omnibus the Human Protein Atlas databases and various other bioinformatic tools, this study investigated the involvement of HSP90B1 in 33 different tumour types. Results: The over-expression of HSP90B1 generally predicted poor overall survival and disease-free survival for patients with tumours, such as adrenocortical carcinoma, bladder urothelial carcinoma, kidney renal papillary cell carcinoma, and lung adenocarcinoma. In this study, HSP90B1 was highly expressed in the majority of tumours. A comparison was made between the phosphorylation of HSP90B1 in normal and primary tumour tissues, and putative functional mechanisms in HSP90B1-mediated oncogenesis were investigated. Additionally, the mutation burden of HSP90B1 in cancer was evaluated along with the survival rate of patients with cancer patients. Conclusion: This first pan-cancer investigation reveals the oncogenic functions of HSP90B1 in various cancers.

## 1. Introduction

It is essential to identify new pan-cancer-related genes and elucidate their functions to have a better understanding of the incredibly complicated process of carcinogenesis. The development of better diagnoses, treatments, and preventive methods for cancer is closely related to a better understanding of genetic changes in tumours [1]. The Cancer Genome Atlas (TCGA) and Gene Expression Omnibus (GEO) databases utilise innovative genomic analysis techniques to obtain a comprehensive understanding of cancer, thereby aiding in generating novel cancer therapies, diagnostics, and preventive strategies [2]. These two databases provide a wealth of publicly available data, providing researchers around the world with invaluable knowledge about the physiological, genetic, and epigenetic profiles of cancer [1]. It also helps researchers screen candidate cancer biomarkers and drug targets, thereby translating cancer genomics into therapeutics and diagnostics and aiding in developing personalised cancer treatments. With the development of the TCGA and GEO databases, there is currently a significant increase in the amount of cancer-related functional genomics datasets of various malignancies, which can be used for an in-depth pan-cancer study [3].

Heat-shock protein 90kDa beta1 (HSP90B1), a stress-inducible molecular chaperone that is a member of the heat-shock protein (HSP) 90 family, is also known as GRP94 and GP96 [4]. Through the maintenance of the endoplasmic reticulum (ER) stress sensors, the preservation of the ER protein folding capability, and the repression of ER-associated proapoptotic machinery, HSP90B1 plays an essential part in regulating the delicate balance between the survival and death of cancer cells [5]. HSP90B1 is crucial for the chaperoning of various proteins, including Toll-like receptors (TLRs) [6], integrin subunits [7], and wnt co-receptor low-density lipoprotein receptor-related protein 6 (LRP6) [8], that have been linked to immune response and cancer development. HSP90B1 mRNA expression has been reported to be elevated in several different cancer tissue types, including breast cancer, oesophageal cancer, glioma tissues, and liver cancer [9,10,11,12]. Various immuno-histochemistry studies report that HSP90B1 protein is significantly overexpressed in a variety of malignancies, including breast, lung, colorectal, oral, oesophageal, and gastric cancers, indicating a connection with cancer development [9,13,14,15,16,17]. Furthermore, HSP90B1 overexpression has been reported to be negatively associated with patient survival in many of the above-mentioned malignancies. For example, patients with breast cancer expressing high levels of HSP90B1 had a significantly lower survival time than those expressing low levels of HSP90B1 [9]. Additionally, HSP90B1 is speculated to be a factor that contributes to bad prognosis in many cancer types, including lung, oesophageal, gastric, and colorectal cancers [13,16,17,18]. Thus, HSP90B1 has a significant relationship with cancer growth and metastases. A previous study reports that administering Her2/neu DNA vaccination to *HSP90B1* HER2+ breast cancer-carrying mice improved immune response against tumours, which was indicated by higher IFN-/IL-4 levels and reduced Tregs at the tumour site [19]. Thus, owing to its ability to activate anti-tumour immune responses on its own, extra-cellular *HSP90B1* has potential as an antigen in tumour vaccines. However, the examination of *HSP90B1* has been limited to a select few forms of cancer, and its significance in other types of tumours remains unknown.

For the first time, to the best of our knowledge, this study aims to comprehensively analyse the role of *HSP90B1* in various cancers. To investigate the potential role of the *HSP90B1* gene as a molecular mechanism in the development of various cancers, *HSP90B1* gene expression, survival status, gene mutations, protein phosphorylation, immune infiltration, and cellular pathways were studied.

## 2. Results

### 2.1. Expression of HSP90B1 in Tumours and Normal Tissues

**Figure 1:**
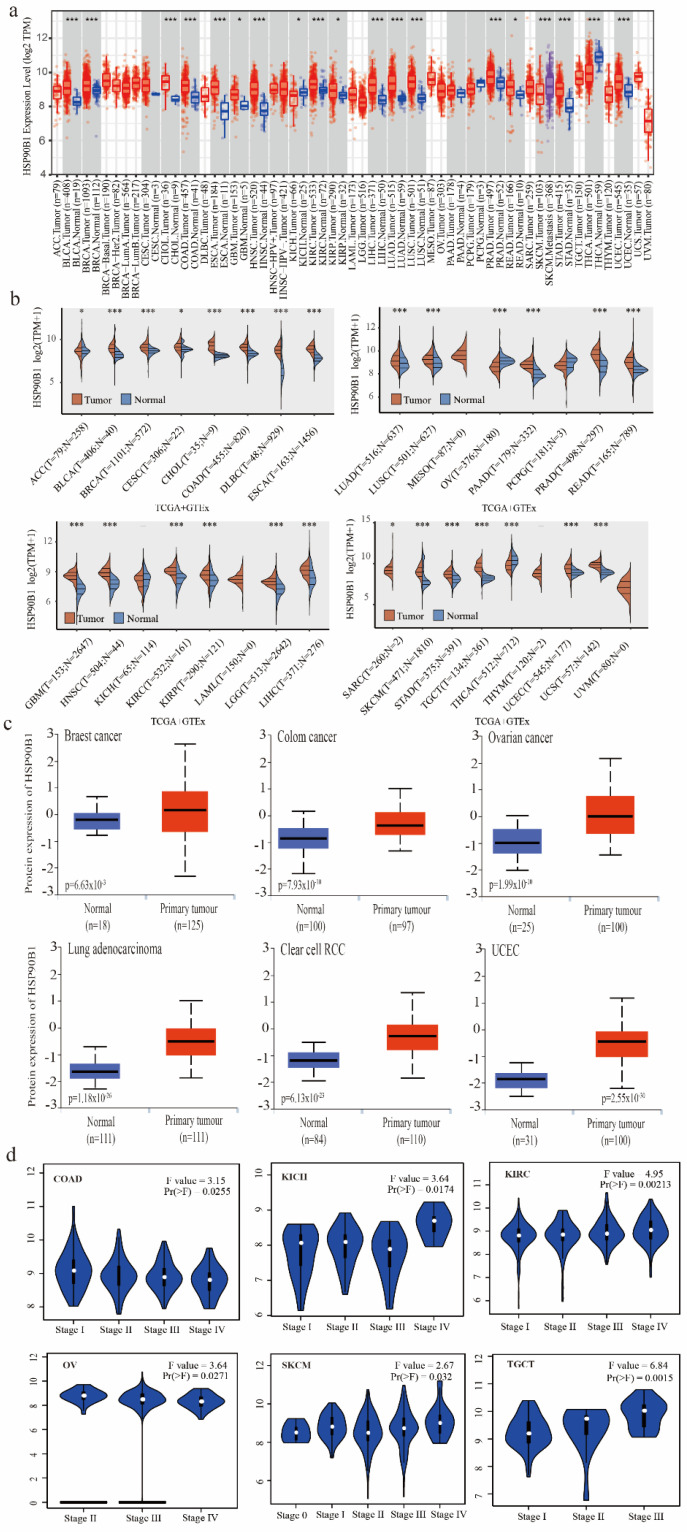
Expression and pathological staging of *HSP90B1* in different tumours. (**a**). Using TIMER2, we examined the levels of HSP90B1 mRNA expression in various malignancies. (**b**) In the TCGA and GTEx datasets, HSP90B1 expression was upregulated in most tumours. (**c**) In the CPTAC dataset, we examined the expression levels of HSP90B1 protein between primary tumour tissues and normal tissues of breast cancer, LUAD, colon cancer, clear cell RCC, UCEC cancer, and ovarian cancer. (**d**) The pathological stages of HSP90B1 in COAD, KICH, KIRC, OV, SKCM, and TGCT were analysed using data from the TCGA database. Logarithmic analysis used Log2 (TPM+1). *p < 0.05; ***p < 0.001.

The TIMER method was used to evaluate the expression patterns of *HSP90B1* in various cancer types, which were obtained from the TCGA database. *HSP90B1* expression was higher in BLCA, BRCA, CHOL, COAD, ESCA, NHSC, KIRC, LIHC, LUAD, LUSC, PRAD, SKCM, STAD, THCA, UCEC (*p* < 0.001), GBM, KICH, KIRP, and READ (*p* < 0.05) than in the corresponding non-tumour tissues (Figure 1a). As shown in Figure 1b, data from the TCGA and GTEx databases were used to analyse the expression of *HSP90B1* in different cancer and normal tissue types. Following this, we performed an analysis of HSP90B1 protein expression in breast cancer, colon cancer, ovarian cancer, lung adenocarcinoma, clear cell RCC, and UCEC using the CPTAC dataset. The expression of HSP90B1 protein in these tumour tissues was observed to be significantly higher than that in normal tissues (*p* < 0.01) (Figure 1c). Furthermore, GEPIA2 analysis revealed that *HSP90B1* expression levels were significantly correlated to the clinical stage of the following cancer types: COAD (*p* = 0.0255), KICH (*p* = 0.0174), KIRC (*p* = 0.00213), OV (*p* = 0.00271), SKCM (*p* = 0.032), and TGCT (*p* = 0.0015) (Figure 1d). Additionally, immunohistochemical staining of the Human Protein Atlas database showed that *HSP90B1* was highly expressed in most malignant tumours (Figure S1). Thus, these findings indicate that *HSP90B1* is significantly expressed in the majority of cancers, which offers a foundation for further investigation.

### 2.2. Prognostic Significance of HSP90B1 in Various Tumours

**Figure 2:**
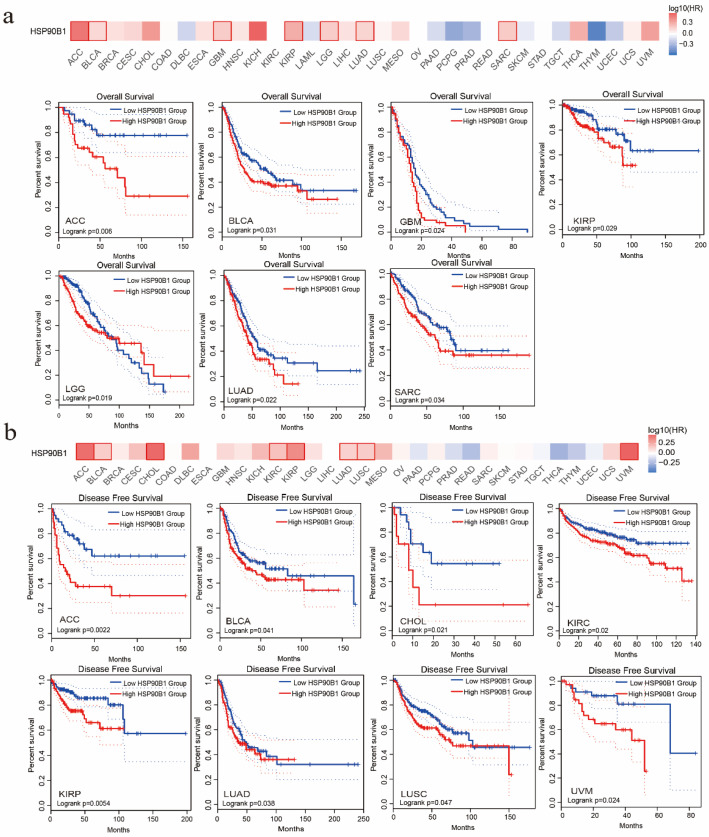
The prognostic relationship of HSP90B1 expression in various tumours. GEPIA2 was used to examine the impact of *HSP90B1* on overall survival (**a**) and disease-free survival (**b**) in various tumour types.

The association between HSP90B1 expression and the patient prognosis was investigated using TCGA datasets. Two groups were formed based on HSP90B1 expression (high- and low-level HSP90B1 expression) to examine the link between HSP90B1 expression and overall patient survival. According to the TCGA datasets, high levels of HSP90B1 expression were linked to poor prognosis for patients with ACC (*p* = 0.006), BLCA (*p* = 0.031), GBM (*p* = 0.024), KIRP (*p* = 0.029), LGG (*p* = 0.019), LUAD (*p* = 0.022), and SARC (*p* = 0.034) (Figure 2a). DFS analysis (Figure 2b) indicated that the high expression of HSP90B1 was associated with poor prognosis of ACC (*p* = 0.0022), BLCA (*p* = 0.041), CHOL (*p* = 0.021), KIRC (*p* = 0.02), KIRP (*p* = 0.0054), LUAD (*p* = 0.038), LUSC (*p* = 0.047), and UVM (*p* = 0.024) tumours. Therefore, tumours with high HSP90B1 expression are speculated to have a bad prognosis.

### 2.3. The Genetic Alteration of HSP90B1

**Figure 3:**
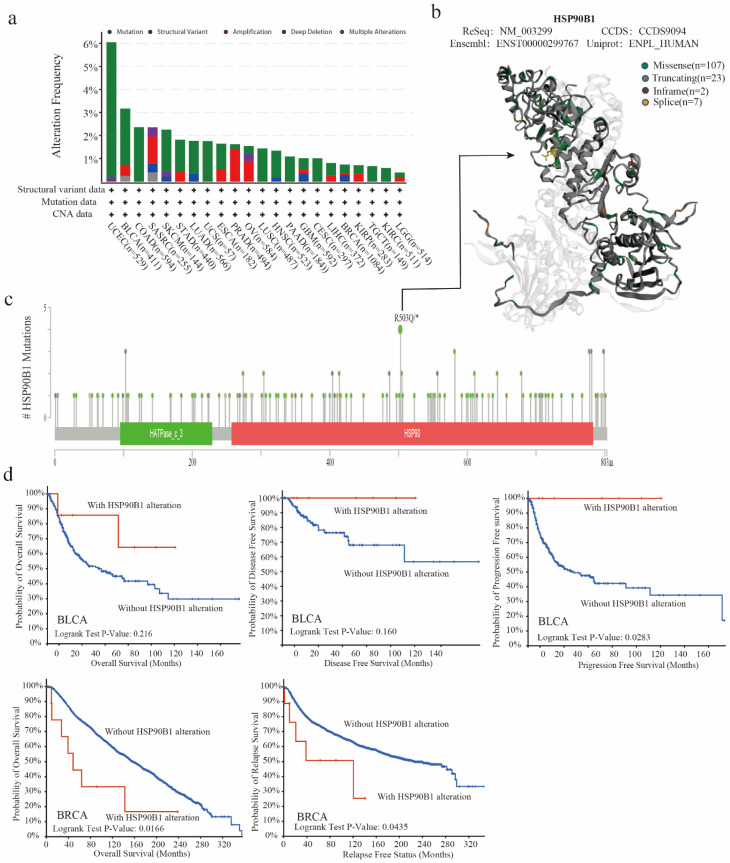
Mutation characteristics of *HSP90B1* in various cancer types. (**a**) Alteration frequency of mutation types and mutation sites of HSP90B1 in different tumours. (**b**,**c**) Different mutation types and the position of the highest mutation site (R503Q/*) in the 3D structure of HSP90B1. (**d**) Potential correlations between BLCA and breast cancer mutational status, overall survival, disease-free survival, progression-free survival, and recurrence-free survival.

The TCGA database was used to examine the genetic changes of HSP90B1 in various tumour samples. As shown in Figure 3a, the highest frequency of HSP90B1 alterations (>6 percent) was observed in patients with UCEC who had a “mutation” as the primary feature, followed by patients with BLCA. Furthermore, Figure 3b displays 145 mutation sites (including 107 missenses, 23 truncating, 2 in frames, and 7 splices) in the 3D structure map using different colours. HSP90B1 acts as a molecular chaperone to promote protein folding by binding to nascent polypeptides, thereby preventing protein misfolding and aggregation. Mutations in genes disrupt this binding, affecting the function of the molecular chaperones. Gene mutations can also alter the role of molecular chaperones in tumours by affecting their sub-localization. Additionally, chaperones act by regulating their client proteins, with mutations affecting the binding of chaperones to their client proteins. Moreover, the missense mutation in *HSP90B1* was observed to be the major type of genetic alteration, with R503Q/* alteration detected in four cases of UCEC (Figure 3c). Furthermore, we hypothesised a possible connection between genetic alterations in HSP90B1 and the clinical survival outcomes of UCEC, BLCA, and breast cancer. Figure 3d shows that HSP90B1-altered patients with BLCA had an improved prognosis in progression-free survival (*p* = 0.0283) but not in overall survival (*p* = 0.216) and disease-free survival (*p* = 0.16) compared with HSP90B1-unaltered cases. In breast cancer, HSP90B1-altered patients showed a significantly worse prognosis in overall survival (*p* = 0.0166) and relapse-free survival (*p* = 0.0435). These findings indicate that mutations in the *HSP90B1* gene could affect the prognosis of individuals with varying malignancies.

### 2.4. Phosphorylation of HSP90B1 Protein was Analysed in Different Tumours

**Figure 4:**
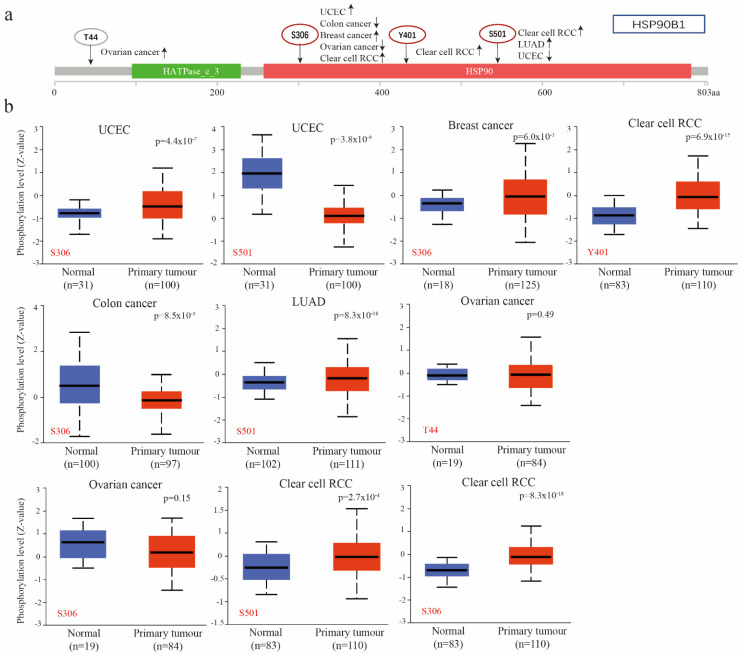
Phosphorylation of *HSP90B1* was analysed in different tumours. (**a**) Four phosphorylated protein sites (T44, S306, Y401, and S501) of HSP90B1 were confirmed using the cBioPortal tool. (**b**) We analysed the differences in HSP90B1 phosphorylated proteins between primary tumour and normal tissues in UCEC, breast cancer, colon cancer, LUAD, ovarian cancer, and clear cell RCC.

Utilizing the CPTAC database, researchers compared the phosphorylation levels of HSP90B1 in normal and primary tumour tissues of patients with UCEC, breast cancer, clear cell RCC, colon cancer, LUAD, and ovarian cancer, revealing significant differences. Figure 4a provides a summary of the HSP90B1 phosphorylation locations and the changes in expression. Figure 4b highlights the difference in HSP90B1 phosphorylation levels between normal and primary tumour tissues. At Y401 site, we found that the phosphorylation level of HSP90B1 in clear cell renal cell carcinoma tissues was higher than that in normal tissues, but there was no significant difference between ovarian cancer tissues at T44 site and corresponding normal tissues.. At S306, the phosphorylation levels of HSP90B1 were higher in UCEC, breast cancer, and clear cell RCC tissues than in normal tissues; however, the opposite effect was observed in colon cancer. Finally, at S501, the phosphorylation levels of HSP90B1 were higher in LUAD and clear cell RCC tissues than in normal tissues and reversed in UCEC. Additionally, the expression of HSP90B1 methylation levels was evaluated in various cancers, revealing that HSP90B1 methylation levels were higher in CHOL and KIRC tumours than in normal tissues, while the opposite was observed in BLCA, COAD, ESCA, HNSC, KIRC, PRAD, and STAD (Figure S2).

### 2.5. Analysis of HSP90B1 Expression and Tumour-Associated Immune Infiltration

**Figure 5:**
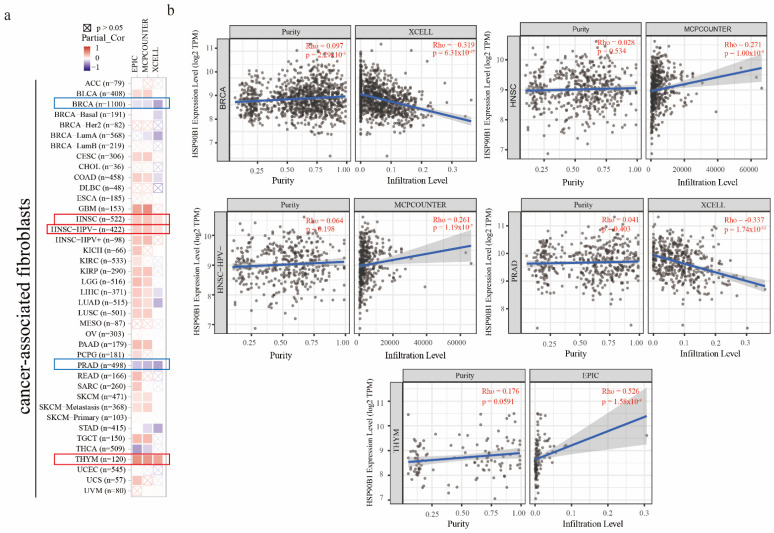
The relationship between immune infiltration of cancer-associated fibroblasts and HSP90B1 expression. (**a**) HSP90B1 expression and the invasion of cancer-associated fibroblasts in various forms of cancer were investigated using the EPIC, MCPCOUNTER, and XCELL algorithms. (**b**) Significant correlation was found between HSP90B and cancer-associated fibroblasts in BRCA, HNSC, HNSC-HPV(-), PRAD, and THYM.

It has been reported that cancer-associated fibroblasts have features that promote tumorigenesis [20]. To investigate the relationship between HSP90B1 expression and cancer-associated fibroblasts, three different algorithms, namely EPIC, MCPCOUNTER, and XCELL, were used. In the TCGA tumours of HNSC, HNSC-HPV(-), and THYM, a statistically positive association between the estimated infiltration value of cancer-associated fibroblasts and HSP90B1 expression was observed. However, a statistically negative correlation was observed between BRCA, PRAD, and HSP90B1 (Figure 5a). Figure 5b displays the scatter plot of the aforementioned tumours, which were generated using an algorithm.

### 2.6. Enrichment Analysis of HSP90B1-Related Genes

**Figure 6:**
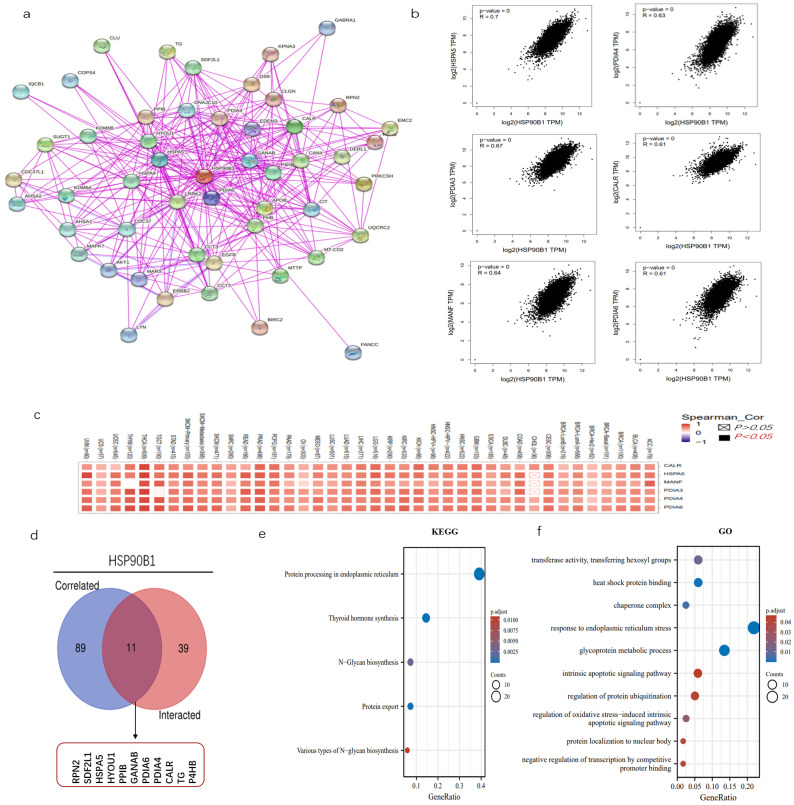
Enrichment analysis of *HSP90B1*. (**a**) We first obtained 50 proteins that could interact with HSP90B1 using the STRING tool. (**b**) Additionally, 100 genes with a high correlation with HSP90B1 were obtained from the TCGA database, and the expression correlation between HSP90B1 and selected targeted genes (including HSPA5, PDIA3, MANF, PDIA4, CALR, and PDIA6) was analysed. (**c**) We analysed the relationship between these six genes and the incidence of each cancer. (**d**) We conducted a crossover analysis of 100 genes with a high correlation with *HSP90B1* and 50 genes that could interact with *HSP90B1*. Enrichment analysis based on *HSP90B1* with high correlation and interaction was performed using KEGG (**e**) and GO (**f**) pathway analyses.

The examination of gene enrichment is an essential part of our research into the molecular mechanisms behind genes that are involved in cancer. STRING was used to perform an analysis on 50 experimentally validated proteins having the potential to interact with HSP90B1, and the resulting interaction network diagram of these proteins is shown in Figure 6a. Then, using the GEPIA2 program, we screened 100 genes that had a high correlation with HSP90B1, revealing six genes that had the greatest connection (Figure 6b). The corresponding heat map further proved that HSP90B1 was positively correlated with the above six genes (HSPA5, PDIA3, MANF, PDIA4, CALR, and PDIA6) in various tumours (Figure 6c). HSPA5, like HSP90B1, also belongs to the heat-shock protein family. Recent studies report that HSPA5 could influence the growth of tumours by controlling ferroptosis [21]. PDIA4 is a potential therapeutic target for the treatment of glioblastoma because it controls the growth of glioblastoma cells by activating the PI3K/AKT/m-TOR pathway and suppressing apoptosis [22]. The combination of CALR and PDIA3 has also been suggested as a possible prognostic biomarker for non-small-cell lung cancer [23], with PDIA6 modulating apoptosis and autophagy of non-small-cell lung cancer cells via the MAP4K1/JNK signalling pathway [24]. Meanwhile, MANF is overexpressed in hepatocellular carcinoma and can be used as a potential diagnostic and prognostic indicator for hepatocellular carcinoma [25]. Subsequently, the genes that could interact with HSP90B1 and those that were highly correlated with HSP90B1 were cross-analysed, and the following 11 genes were obtained: RPN2, SDF2L1, HSPA5, HYOU1, PPIB, GANAB, PDIA6, PDIA4, CALR, TG, and P4HB (Figure 6d). Furthermore, KEGG data showed that “thyroid hormone synthesis” and “protein processing in endoplasmic reticulum” could be involved in the effect of HSP90B1 on tumour pathogenesis (Figure 6e). Then, GO enrichment analysis further indicates the mechanism of HSP90B1 regulating tumour progression (Figure 6f).

### 2.7. Expression of HSP90B1 in Some Tumours

**Figure 7:**
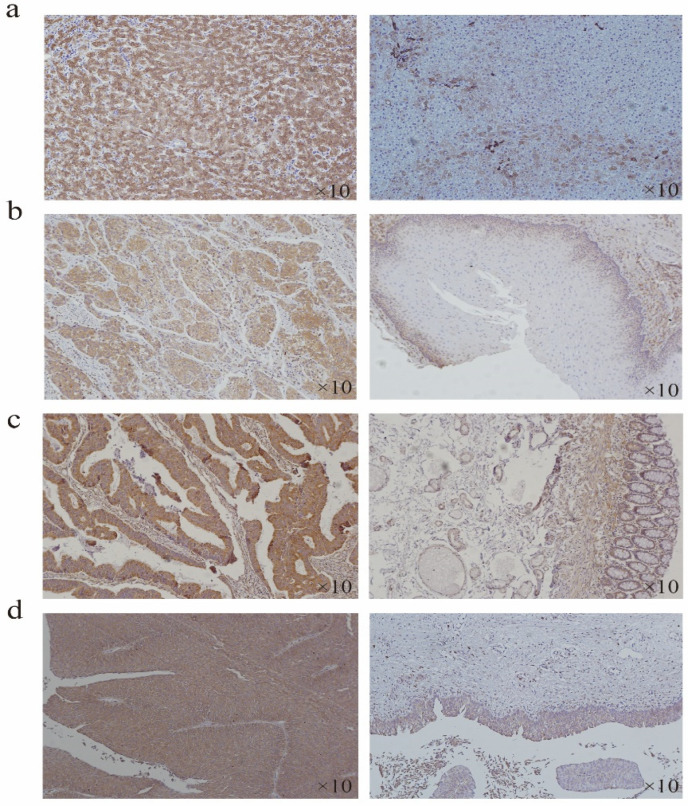
Expression of HSP90B1 in liver cancer, oesophageal cancer, colorectal cancer, and bladder cancer. Immunohistochemistry was used to analyse the expression of HSP90B1 in liver cancer (**a**), oesophageal cancer (**b**), colorectal cancer (**c**), and bladder cancer (**d**).

According to the expression difference of HSP90B1 RNA in tumours and corresponding normal tissues (Figure 1a), the tumour type with the most significant difference was selected for immunohistochemistry (IHC) analysis. The results showed that the staining intensity of HSP90B1 protein in liver cancer, oesophageal cancer, colorectal cancer, and bladder cancer was higher than that in the corresponding normal tissues. Moreover, HSP90B1 protein was mainly expressed in the cytoplasm and not in the nucleus (Figure 7a-d).

## 3. Discussion

Cancer is a global public health issue and one of the primary causes of high morbidity and mortality [26]. Current approaches for treating cancer include surgical removal of the tumour, radiation therapy, chemotherapy, and targeted therapy. Despite advances in the treatments of cancer, there remain a significant proportion of people who do not benefit from them. This conundrum underlines the need to comprehensively understand the pathways that lead to the development of tumours [27]. Therefore, it is necessary to identify additional targets and biomarkers that could be used in the diagnosis and treatment of cancer [28]. In the field of precision medicine, discovering reliable cancer biomarkers and investigating their function in disease progression could aid in the development of cancer treatments.

HSPs are a category of stress proteins that are very well-conserved and found in a wide variety of species. In its role as a molecular chaperone, it plays a primary role in the assembly and sorting, transmembrane transport, and degradation of intracellular proteins. These proteins are induced in response to a variety of stresses, including traumatic injury, infectious diseases, hypoxia, and malignant tumours [29]. Ischemia and hypoxia are typically present in tumour tissues, consequently generating a quick stress response and increasing the expression level of HSPs. Previous studies report a connection between HSP90B1 and various illnesses, including cancer, pneumonia [30], polycystic ovary syndrome [31], ulcerative colitis [32], and others. Notably, an emphasis on the function of HSP90B1 in tumour development is currently observed. Furthermore, recent studies report that the aberrant expression of HSP90B1 in lung adenocarcinoma [13], bladder cancer [33], colorectal cancer [34], and tongue squamous cell carcinoma [35] is associated with a poor prognosis. Additionally, an internalized antibody targeting cell surface HSP90B1 was also shown to effectively inhibit tumour angiogenesis in colorectal cancer [36]. HSP90B1 can also affect the progression of thyroid cancer by regulating the expression and sub-localization of its client protein ITGA2 [37]. However, these studies are limited to some tumours, and thus, more studies are required to investigate their role in other tumours.

The overexpression of HSP90B1 on the cell membrane has been reported to boost cell proliferation and tumour development by increasing HER2 dimerization and the downstream signalling cascade. Moreover, HSP90B1 also interacts specifically with HER2 on the cytoplasmic membrane of human breast cancers [38]. Additionally, it has been shown that HSP90B1 induces apoptosis in prostate cancer cells, thereby preventing the cells from migrating [39]. Collectively, these studies suggest that cell surface HSP90B1 has potential as a therapeutic target for cancer treatment.

Despite the increasing number of research devoted to the investigation of the role of HSP90B1 in many disorders, including cancer, it remains unclear whether HSP90B1 plays a pro-oncogenic or anti-oncogenic role in tumour pathogenesis. Using the TCGA database, the current study’s all-encompassing method comprised an investigation of the *HSP90B1* expression level in 33 different cancers. Additionally, we used the CPTAC and GEO databases to methodically gather and combine data on proteins and phosphor proteins in addition to other molecular characteristics and genetic changes.

On examining the levels of HSP90B1 expression in each tumour, it was observed to be highly expressed in the majority of cancers. The variations in HSP90B1 expression levels observed across the spectrum of tumour types are indicative of its diverse processes and functions. Furthermore, GEPIA2 revealed that poor overall survival in ACC, BLCA, GBM, KIRP, LGG, LUAD, and SARC was significantly associated with high HSP90B1 expression; however, in ACC, BLCA, CHOL, KIRC, KIRP, LUAD, LUSC, and UVM, poor disease-free survival was significantly correlated with high HSP90B1 expression. Based on these findings, HSP90B1 has potential as a biomarker for determining the outlook for patients with cancer. The investigation of the mutation driving mechanism revealed that the HSP90B1 gene alterations in UCEC were mostly missense mutations, while the sarcoma mutations were amplification mutations. Additionally, the mutation of *HSP90B1* showed an important effect on the progression-free survival of patients with BLCA patients and the overall survival and relapse-free survival of patients with breast cancer.

An important step in the development of cancer is the phosphorylation–dephosphorylation cascade. By determining the amount of total protein and phosphorylated protein, the probable molecular pathways of HSP90B1 in UCEC, breast cancer, blear cell RCC, Colon cancer, LUAD, and ovarian cancer can be elucidated. Accordingly, the phosphorylation sites of HSP90B1 were significantly increased in breast cancer, LUAD, and clear cell RCC but significantly reduced in colon cancer. However, contradictory results were obtained at several locations for UCEC and ovarian cancer, suggesting that these cancers may have distinct causes. Nonetheless, studies on the correlation between HSP90B1 phosphorylation and tumorigenesis are insufficient and require further analyses. This study identified four novel phosphorylation sites, which could be one of the important pathways through which HSP90B1 functions in tumours. Subsequently, HSP90B1 expression and the immune invasion of cancer-associated fibroblasts in various tumours were analysed. In BRCA and PRAD, HSP90B1 expression was negatively correlated with tumour-associated fibroblast infiltration, while in HNSC and THYM, HSP90B1 expression was positively correlated with tumour-associated fibroblast infiltration.

Additionally, we also identified 11 genes that interacted with HSP90B1 and were highly correlated with HSP90B1: RPN2, SDF2L1, HSPA5, HYOU1, PPIB, GANAB, PDIA6, PDIA4, CALR, TG, and P4HB. RPN2 is speculated to be involved in the progression of malignant tumours by regulating various signalling pathways, such as STAT3, NF-κB, and PI3K-Akt [40,41]. It is also considered a promising prognostic biomarker for colorectal cancer, non-small-cell lung cancer, and gastric adenocarcinoma [42,43,44]. Furthermore, SDF2L1 mRNA is significantly upregulated by the unfolded protein response (UPR) and can interact with ER chaperone complexes, including HSPA5, to regulate chaperone activity. [45]. Thus, there exists a high possibility that SDF2L1 interacts with HSP90B1, which can be explored in our future studies. Chen et al. found that multiple stemness-related markers were downregulated on HSPA5 knockdown and demonstrated that HSPA5 is a chaperone protein associated with cancer stemness maintenance in head and neck cancer cells [46]. In addition to head and neck cancer, a potential function of GRP78 in cancer stemness has also been reported in breast cancer and glioma [47,48]. HYOU1 is a chaperone protein located in the ER and serves not only as a potential therapeutic target for cancer but also as an immunostimulatory adjuvant, owing to its anti-tumour immune response. It is also used as a molecular target for the treatment of many ER-related diseases [49]. PPIB induces chemoresistance in colorectal cancer by degrading wild-type p53 [50], and it can regulate hepatocellular carcinoma cell apoptosis and metastasis [51]. Currently, research on the GANAB gene in cancer is scarce. PDIA6 has been shown to promote pancreatic cancer progression and immune evasion through β-catenin and PD-L1 deubiquitination [52]. It can also affect the progression of bladder cancer, oral squamous cell carcinoma, and non-small-cell lung cancer [24,53,54]. PDIA4 is a novel ER stress chaperone that regulates adiponectin expression and inflammation in adipose tissue [55], and it is also involved in tumour progression by affecting apoptosis [56]. Furthermore, PDIA4 and PDIA6 regulate cisplatin-induced lung adenocarcinoma cell death resistance [57]. CALR can not only serve as a potential prognostic biomarker for lung cancer [58] but is also an important target for tumour immunotherapy [59]. The *TG* gene mainly plays a role in thyroid diseases, with very few studies being reported on this topic. P4HB is also a chaperone protein that has been associated with temozolomide resistance via UPR in gliomas [60]. Therefore, these 11 genes can regulate tumour progression in various ways; however, further experimental analysis is required to prove this conclusion. Furthermore, GO and KEGG analyses suggest that HSP90B1 could regulate the occurrence and development of tumours via various mechanisms, such as participating in ER stress response or regulating apoptosis. 

## 4. Conclusions

Our comprehensive pan-cancer analysis of HSP90B1 revealed that the high expression of HSP90B1 was associated with the poor clinical prognosis of various human cancers, indicating that HSP90B1 can be used as an effective biomarker in cancer. Additionally, a statistical correlation was observed between the expression of HSP90B1 and cancer-related fibroblast infiltration, tumour mutation burden, and protein phosphorylation, which validated the role of HSP90B1 in tumorigenesis from various perspectives. However, this study has some limitations. Most of our research is based on data from online databases. Thus, more in vitro and in vivo experiments are required to verify our analyses.

## 5. Materials and methods

### 5.1. Gene Expression Analysis

We compared HSP90B1 mRNA expression between tumour and normal tissues, which were obtained from the TCGA database, using TIMER2 (http://timer.cistrome.org/ (accessed on 21 July 2022)-> Gene DE). Moreover, data from the GTEx and TCGA databases were used to examine the expression of HSP90B1 in different types of cancer and normal tissues. Furthermore, the difference in HSP90B1 protein expression between different tumour types and corresponding adjacent tissues was analysed using UALCAN [21] (http://ualcan.path.uab.edu/analysis -prot.html (accessed on 25 July 2022)->CPTAC analysis) and the CPTAC (Clinical Proteomic Tumour Analysis Consortium) dataset. HSP90B1 expression in different cancers, which were obtained from the Human Protein Atlas database, was analysed using immunohistochemical labelling. Moreover, the “Stage Plot” module of GEPIA2 (http://gepia2.cancer-pku.cn/ (accessed on 28 July 2022)) was used to examine the expression levels of HSP90B1 in distinct cancer types at different clinical stages.

### 5.2. Survival Prognosis Analysis

The overall survival (OS) and disease-free survival (DFS) were obtained for HSP90B1 in all TCGA tumours using the “survival map” module of GEPIA2.

### 5.3. Genetic Alteration Analysis

cBioPortal for Cancer Genomics (http://www.cbioportal.org (accessed on 21 July 2022)) was used to evaluate the genetic alterations of HSP90B1 in pan-cancer, including somatic mutations, structural variants, amplifications, profound deletions, and multiple alterations. Moreover, in TCGA patients with and without HSP90B1 gene mutation, the data on OS, DFS, PFS, and RFS were obtained.

### 5.4. Immuno-Infiltration Analysis

The association of HSP90B1 expression to immune invasion in all TCGA cancers was determined using the “Immune genes” module of TIMER2, which also allowed the selection of CD8+ T cells and cancer-associated fibroblasts from the immune system. Estimates of immune infiltration were made using the EPIC, MCPCOUNTER, and XCELL algorithms. *p*-values and partial correlation values were calculated using the purity-corrected Spearman rank correlation test.

### 5.5. Functional Enrichment Analysis

A search for “HSP90B1” and “Homo sapiens” in the STRING database was the first step in this analysis, and then, the following parameters were set: Network edges (“evidence”), the maximum number of participants to display (“no more than 50 participants”), and active interactions (“experiments”) were included in the “Low confidence (0.150)” minimum score requirement. Subsequently, 50 proteins were obtained that could bind to HSP90B1. GEPIA2 was used to analyse the 100 genes with the highest correlation with HSP90B1. Finally, HSP90B1-binding and HSP90B1-interacting genes were compared using the interactive Venn diagram viewer Jvenn. Additionally, the two sets of data were combined to perform KEGG and GO pathway analysis.

### 5.6. Immunohistochemistry

Antibodies for HSP90B1 (ab238126) were purchased from Abcam company. Samples were embedded in paraffin at a thickness of 4 μm. Deparaffinization and rehydration were performed on each slide. To eliminate aldehyde linkages from antigens, they were reextracted using a pressure cooker and 0.01 M citrate buffer (pH 6). The slides were incubated with HSP90B1 antibody (1: 2000) overnight. After incubating the HRP-labelled secondary antibody for 1 h, immunodetection was performed the following day using diaminobenzidine, according to the manufacturer’s instructions [61].

### 5.7. Statistical Analysis

*t*-Tests were used to evaluate differences in HSPA5 expression between cancer and normal tissues. Univariate Cox regression analysis was used to obtain the HR and *p*-value for the survival analysis. Patients were divided into two groups, with high and low HSPA5 expression, respectively, and Kaplan–Meier analysis was utilized to compare their survival rates. All statistical tests were conducted at the *p* < 0.05 significance level. * *p* < 0.05, ** *p* < 0.01, *** *p* < 0.001.

## Data Availability

The datasets obtained from TCGA database (https://portal.gdc.cancer.gov/ accessed on 25 July 2022) and UALCAN database(http://ualcan.path.uab.edu/analysis.html accessed on 25 July 2022), partial analysis by Cbioportal for Cancer Genomics website (http://www.cbioportal.org accessed on 25 July 2022) and TIMER2.0 database (http://timer.cistrome.org/ accessed on 25 July 2022).

## Supplementary Materials

The following are available online at https://www.mdpi.com/article/10.3390/biom12101377/s1, Figure S1: Expression of HSP90B1 protein in various tumour tissues; Figure S2: The methylation expression level of HSP90B1 in various cancers and corresponding normal tissues.

## Abbreviations

ACC	Adrenocortical carcinoma
BLCA	Bladder urothelial carcinoma
BRCA	Breast invasive carcinoma
CESC	Cervical squamous cell carcinoma and endocervical adenocarcinoma
CHOL	Cholangiocarcinoma
COAD	Colon adenocarcinoma
DLBC	Lymphoid neoplasm diffuse large B-cell lymphoma
ESCA	Oesophageal carcinoma
GBM	Glioblastoma multiforme
HNSC	Head and neck squamous cell carcinoma
KICH	Kidney chromophobe
KIRC	Kidney renal clear cell carcinoma
KIRP	Kidney renal papillary cell carcinoma
LAML	Acute myeloid leukaemia
LGG	Brain lower-grade glioma
LIHC	Liver hepatocellular carcinoma
LUAD	Lung adenocarcinoma
LUSC	Lung squamous cell carcinoma
MESO	Mesothelioma
OV	Ovarian serous cystadenocarcinoma
PAAD	Pancreatic adenocarcinoma
PCPG	Pheochromocytoma and paraganglioma
PRAD	Prostate adenocarcinoma
READ	Rectum adenocarcinoma
SARC	Sarcoma
SKCM	Skin cutaneous melanoma
STAD	Stomach adenocarcinoma
STES	Stomach and oesophageal carcinoma
TGCT	Testicular germ cell tumours
THCA	Thyroid carcinoma
THYM	Thymoma
UCEC	Uterine corpus endometrial carcinoma
UCS	Uterine carcinosarcoma
UVM	Uveal melanoma
