# Supplementary material for: A Pan-Cancer Analysis of Heat-Shock Protein 90 Beta1(HSP90B1) in Human Tumours
Source: Biomolecules. 2022 Sep 26;12(10):1377. doi: 10.3390/biom12101377 (PMC9599833; doi:10.3390/biom12101377)
Supplement: Supplementary file 1 [file biomolecules-12-01377-s001.zip › biomolecules-1909432-supplementary.pdf]

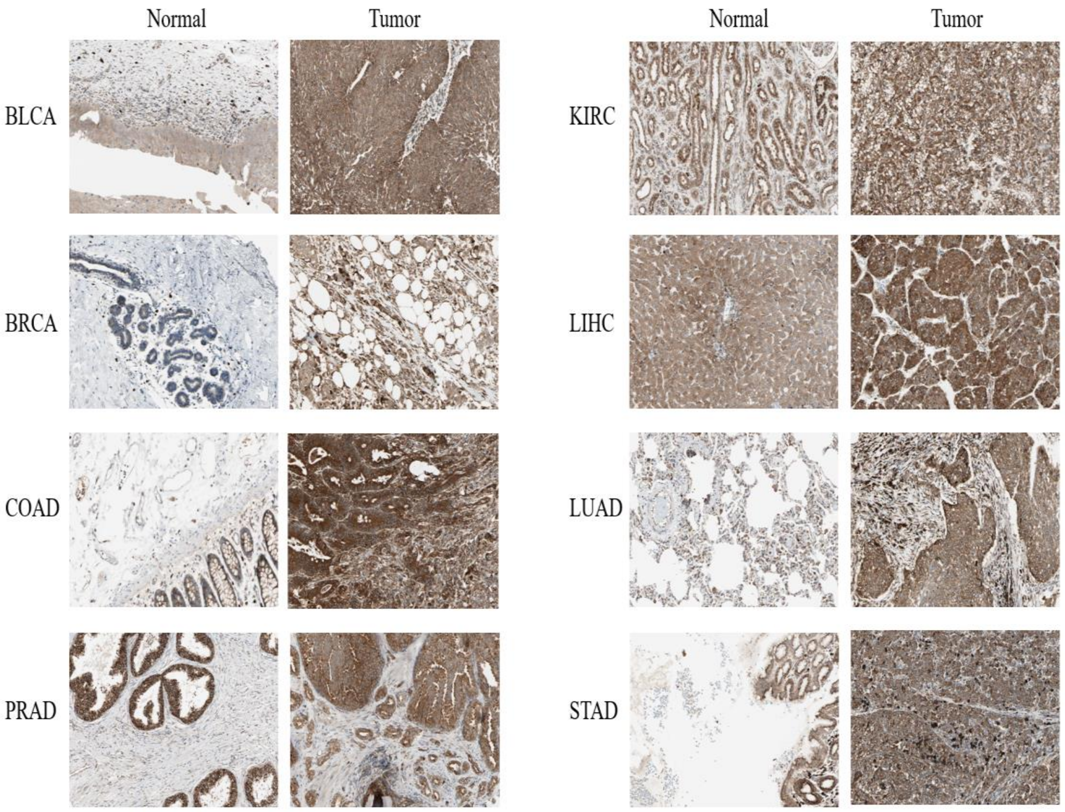

Figure S1. Expression of HSP90B1 protein in various tumor tissues.

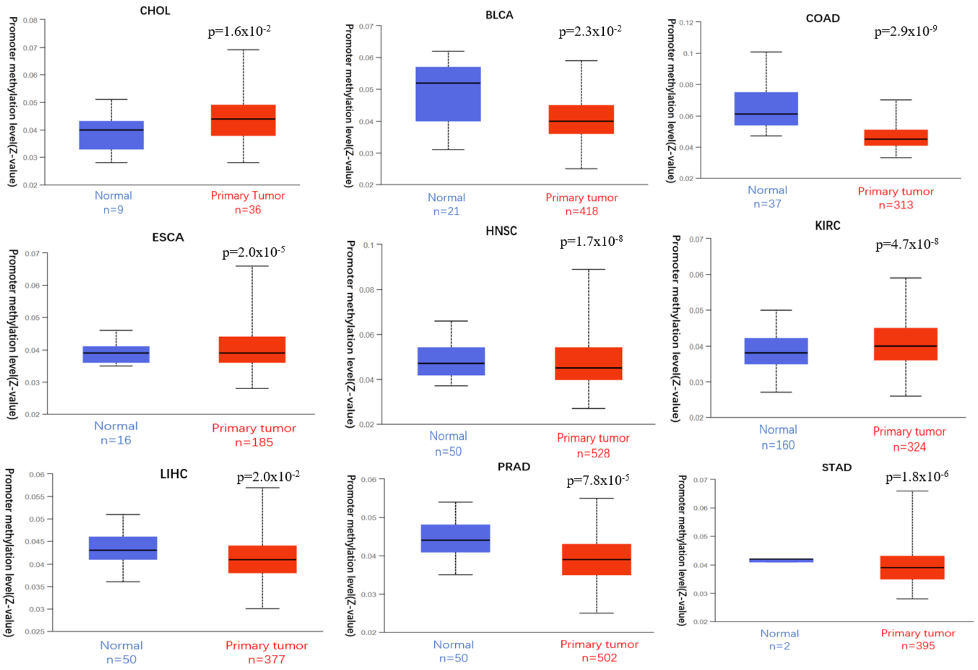

Figure S2. The methylation expression level of HSP90B1 in various cancers and corresponding normal tissues.
